# Persistent SARS-CoV-2 infection in patients with B-cell deficiency: a case series of successful antiviral treatment of four patients

**DOI:** 10.48101/ujms.v128.9807

**Published:** 2023-10-11

**Authors:** Lisa Faxén, Marie Edvinsson

**Affiliations:** Department of Medical Sciences, Section of Infectious Diseases, Uppsala University Hospital, Uppsala, Sweden

**Keywords:** COVID-19, anti-CD20, rituximab, epcoritamab, B-cell depletion, B-cell deficiency, persistent infection, immunosuppression

## Abstract

Persistent severe acute respiratory syndrome coronavirus 2 (SARS-CoV-2) infection in immunocompromised patients remains a major medical challenge. Diagnosing the syndrome is difficult as symptoms may mimic other diseases and treatment guidelines are lacking. We describe a case series of four patients with persistent SARS-CoV-2 infection that all had an underlying B-cell deficiency due to rituximab treatment (in one case in combination with epcoritamab). In all four patients, it was initially difficult to recognize the persistent disease, leading to a duration of illness between 45 and 242 days. Two patients were only positive for SARS-CoV-2 by polymerase chain reaction (PCR) in the nasopharynx at the beginning of the disease but were later repeatedly negative. However, when bronchoalveolar lavage was performed, a positive SARS-CoV-2 PCR was revealed from the lower airways in both patients. The difficulties establishing diagnosis contributed to these two patients’ long disease course. The longest disease duration was in the patient treated with rituximab and epcoritamab, who also responded poorly to single standard antiviral treatment. This patient ultimately cleared the infection after administering a combination treatment with remdesivir and nirmatrelvir/ritonavir. After a confirmed diagnosis, the other three patients cleared the infection when they were finally treated with antivirals. Increasing clinicians’ awareness of this condition is important as it might be treatable once diagnosed. Further studies are warranted to define the condition and treatment strategy with greater precision.

## Introduction

Persistent severe acute respiratory syndrome coronavirus 2 (SARS-CoV-2) infection in immunocompromised patients is a medical challenge for which treatment strategies are yet to be defined. Moreover, the condition may be complex for the treating physicians to recognize as symptoms mimic other diseases such as bacterial pneumonia or cryptogenic organized pneumonia. Therefore, physicians must consider this condition when assessing immunocompromised patients with respiratory symptoms that are or previously have been positive for SARS-CoV-2.

An important risk factor for protracted SARS-CoV-2 infection is B-cell depletion (1, 2), where continuing polymerase chain reaction (PCR) positivity from the nasopharynx and bronchoalveolar lavage fluid (BALF) has been demonstrated (3). A common immunosuppressive therapy affecting B-cell function is the monoclonal antibody against the B-cell marker CD20, rituximab (4). This therapy is used against different disorders (e.g. autoimmune diseases, B-cell malignancies, and multiple sclerosis) (4). One consequence of the treatment is often a reduced response to vaccines, which is also the case regarding vaccines against SARS-CoV-2 (5, 6). Another treatment affecting B-cells is the bispecific antibody epcoritamab, which exerts its effect on B-cells by T-cell–mediated cytotoxicity (7). This antibody is thus far mainly used in treating different types of lymphoma. The disease course of SARS-CoV-2 infection in patients treated with epcoritamab has not been thoroughly investigated.

Antiviral treatment of SARS-CoV-2 infection is recommended for patients more likely to become severely ill, including those with immunosuppressive therapy. The antiviral treatment is recommended to be administered within the first week of symptoms. However, not all patients seek medical care if they only have mild symptoms. Some patients with B-cell dysfunction may have a prolonged condition with intermittent respiratory and systemic symptoms without clearing the infection (8, 9). Guidelines on treating these patients late in the course of the disease are lacking and clinicians can only rely on hitherto published case reports for guidance. In 2022, at the Infectious Disease Clinic, Uppsala University Hospital, Uppsala, Sweden, we treated four patients suffering from a prolonged SARS-CoV-2 infection. The four patients had an underlying medical condition that was treated with rituximab. For these patients, it took several weeks to recognize their condition as a persistent SARS-CoV-2 infection. Fortunately, the infection was cleared after antiviral treatment. One patient (Case 3) was treated with a more experimental approach after a literature search, where a case report suggested combination treatment (10). We want to share the story of our four patients and how we treated them to increase awareness of this condition among other clinicians.

## Patients

**Figure 1:**
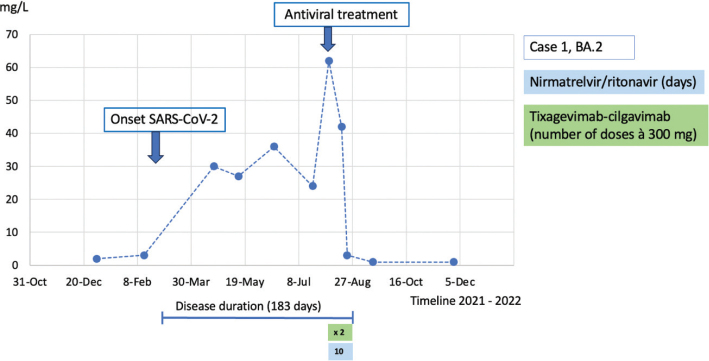
Case 1: CRP course during the disease duration and antiviral treatment.

**Figure 2:**
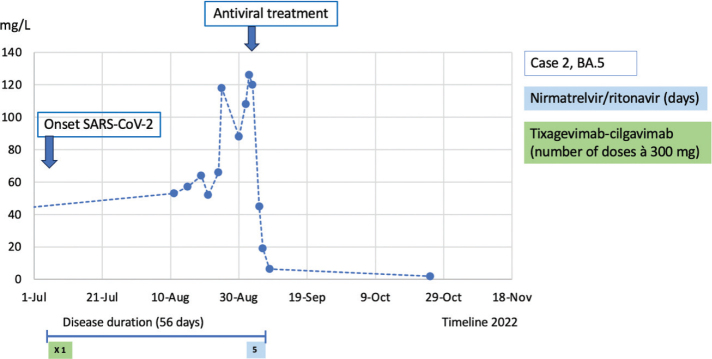
Case 2: CRP course during the disease duration and antiviral treatment.

**Figure 3:**
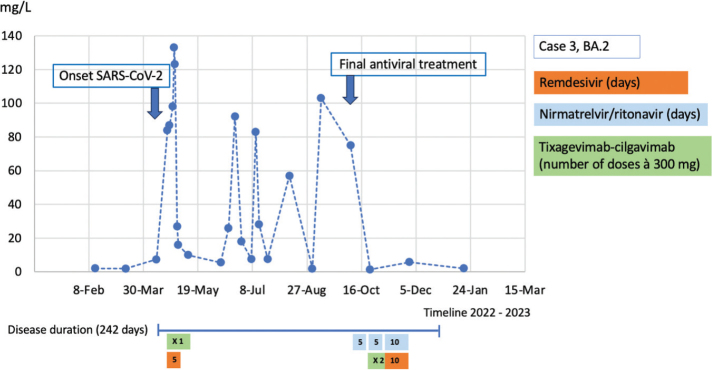
Case 3: CRP course during the disease duration and antiviral treatment.

**Figure 4:**
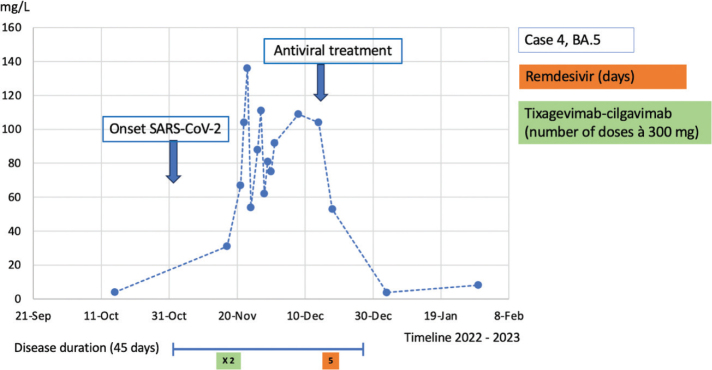
Case 4: CRP course during the disease duration and antiviral treatment.

In the present paper, we describe four patients with B-cell–depleting therapies that developed persistent SARS-CoV-2 infection and were successfully treated after tailored antiviral treatment. All patients were cared for at the Infectious Disease Clinic, Uppsala University Hospital, Uppsala, Sweden, from April to December 2022. This is a tertiary care hospital, including an Infectious Disease unit, an Oncology unit, a Rheumatology unit, and a Transplantation unit for kidney transplantation. Written informed consent for publication was obtained for all cases. Patients are described as follows, and some parameters are summarized in Table 1 and Figures 1–4. Apart from repeated sampling for SARS-CoV-2, all patients were thoroughly investigated for other microbial pathogens and disease causes during their symptomatic period. Only a few tests were positive. These findings are presented as follows in the case presentations. As the aim of this case series is to describe the cases, no selection of patients with different clinical outcomes has been performed. To date, we have not encountered any cases with persistent SARS-CoV-2 infection that has not responded to tailored antiviral treatment after recognition of the condition.

**Table 1:** Previous immunosuppressive treatment, disease duration, SARS-CoV-2 variant, and antiviral and antibiotic treatment in the four described cases.

Clinical data	Patient 1, 25-year-old female	Patient 2, 54-year-old female	Patient 3, 76-year-old male	Patient 4, 52-year-old female
Immunosuppressive treatment (days since last dose before onset of disease)	Rituximab (3 days) Prednisolone	Rituximab (209 days) Leflunomide	Rituximab (1 day) Leflunomide Epcoritamab (1 day)	Rituximab (136 days) Prednisolone Cyclosporine
Disease duration before first treatment with antivirals	178 days	50 days	12 days	40 days
SARS-CoV-2 variant	BA.2	BA.5	BA.2	BA.5
Highest level of CRP in mg/L, normal range <5 mg/L (day of disease)	62 (day 162)	126 (day 50)	133 (day 16) 103 (day 151)	136 (day 21)
Lowest value of lymphocyte count (normal range 0.7–3.9 × 10^9^/L) (day of disease)	0.6 (day 151)	0.5 (day 50)	1.2 (day 12)	0.1 (day 25)
Lowest value of neutrophil count (normal range 1.3–5.4 × 10^9^/L) (day of disease)	6.2 (day 151)	2.7 (day 37)	0.6 (day 77)	2.2 (day 27)
Treatment with gamma globulin	No	No	No	No
Total disease duration	183 days	56 days	242 days	45 days
Antibiotic treatment	- Doxycycline - Doxycycline - Amoxicillin - Co-trimoxazole	- Moxifloxacin - Meropenem	- Piperacillin-tazobactam - Meropenem - Amoxicillin/clavulanic acid - Doxycycline - Clindamycin	- Piperacillin-tazobactam - Amoxicillin/clavulanic acid

## Case 1

**Figure 5:**
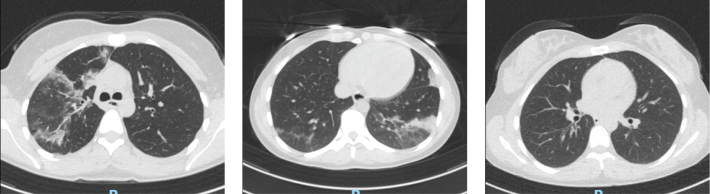
CT scans of Case 1 in (a) June, (b) August, and (c) October, demonstrating a pattern interpreted as organizing pneumonia that initially progressed and then regressed after antiviral treatment.

Case 1 was a 25-year-old female with mixed connective tissue disease (MCTD). She was treated with rituximab every 6th month and prednisolone 5 mg daily. The patient had received three doses of the SARS-CoV-2 mRNA vaccine but had not responded serologically. In February 2022, she developed a fever and cough and tested positive for SARS-CoV-2 by PCR. She experienced a short clinical improvement after 14 days of symptoms, but after a couple of days, the symptoms returned. After 8 weeks of persistent symptoms with fluctuating fever, cough, and dyspnea, she was assessed at the Infectious Disease Clinic. The PCR for SARS-CoV-2 was positive in the nasopharynx but negative in blood. SARS-CoV-2 serology was negative. C-reactive protein (CRP) was 30 mg/L (normal range <5 mg/L). She did not require supplemental oxygen. A chest computed tomographic (CT) scan revealed an image consistent with atypical pneumonia, and because the nasopharyngeal culture was positive for *Haemophilus influenzae*, she was treated with doxycycline against suspected pneumonia. No treatment against SARS-CoV-2 was prescribed. She experienced 1 week of symptomatic improvement, but then the fluctuating fever, cough, and dyspnea returned and persisted. During the following 4 months, she was reassessed several times at the Infectious Disease Clinic (May, June, July, and August), and every time she tested negative by PCR for SARS-CoV-2 in the nasopharynx. CRP was approximately 30 mg/L. During the first three assessments, the nasopharyngeal culture was positive for *Haemophilus influenzae*, resulting in antibiotic treatment on every occasion (doxycycline, amoxicillin, and co-trimoxazole) with no symptomatic improvement. Immunoglobulins in serum were tested with a normal IgG at 8.0 (normal range 6.7–14.5), a low IgM at 0.15 (normal range 0.27–2.1), and a low IgA at <0.05 (normal range 0.88–4.5). As she had not had any problems with infections earlier, she had no history of immunoglobulin treatment. A new chest CT in June showed ground-glass opacities and multilobar consolidation. This image was consistent with a pattern of organizing pneumonia (OP) (Figure 5a), which gave rise to interdisciplinary discussions on whether this was an infectious condition or an interstitial lung disease associated with her MCTD (MCTD-ILD). The patient was planned for a follow-up CT scan after 6–8 weeks. In August, she was again assessed at the Infectious Disease Clinic because of persistent symptoms of high fever, effort dyspnea, cough, and fatigue. CRP was now 62 mg/L, and a new chest CT scan revealed progress of OP (Figure 5b). She was admitted to the hospital and underwent a bronchoalveolar lavage (BAL) that surprisingly showed a positive PCR for SARS-CoV-2. The cycle threshold (Ct) value was 31.3, and the strain was typed to BA.2 (the predominant variant in February 2022), strongly suggesting persistent SARS-CoV-2 infection rather than reinfection. The PCR in the nasopharynx remained negative. The patient was treated with nirmatrelvir/ritonavir (Paxlovid) for 10 days and a double dose (300 + 300 mg) of tixagevimab-cilgavimab (Evusheld). After 5 days of treatment, CRP was normal (Figure 1), and the patient felt relief from the clinical symptoms. After that, she gradually recovered fully. A follow-up CT scan was performed 2 months later, showing regression of earlier pulmonary infiltrates (Figure 5c), and the patient self-reported resolution of all symptoms. This recovery remained on the latest follow-up, 5 months after discharge.

## Case 2

Case 2 was a 54-year-old female suffering from rheumatoid arthritis and was treated with rituximab every 6th month and leflunomide 20 mg daily. She had received five doses of the SARS-CoV-2 vaccine but had not responded serologically. Immunoglobulins in serum had been tested before with a reduced IgG at 3.7 (normal range 6.7–14.5) and IgM at 0.17 (normal range 0.27–2.1) but normal IgA. As she had not had any problems with infections, she was not on any immunoglobulin treatment. In July 2022, she developed a fever and tested positive for SARS-CoV-2 by PCR. She was assessed at the Infectious Disease Clinic where she had stable vital signs and received 300 mg tixagevimab-cilgavimab. The PCR in blood was negative for SARS-CoV-2, and the strain in the nasopharynx was later typed to BA.5. At that time, nirmatrelvir/ritonavir was not yet available in Sweden. She improved for a few weeks, but the fever returned in August. The patient was repeatedly assessed at the Infectious Disease Clinic, where she presented with fever and cough. CRP was consistently around 50, alkaline phosphatase (ALP) around 6–7 u/L (normal range 0.60–1.8 u/L), alanine transaminase (ALT) (ALT) around 0.9–2 u/L (normal range 0.15–0.75 u/L), and every time with stable vital signs. PCR for SARS-CoV-2 in the nasopharynx was repeatedly negative. After 2 weeks, she was admitted to the hospital due to persistent and increasing symptoms. PCR for SARS-CoV-2 in the nasopharynx was still negative. Ultrasound of the liver was normal, but liver function tests continued to increase slightly. A chest CT scan revealed infiltrates indicating atypical pneumonia. A BAL was performed for which the fluid initially only tested positive for *Haemophilus influenzae* with PCR. Moxifloxacin was then prescribed, and the patient was discharged as she wanted to return home and had stable vital signs; however, the fever was still present. On the planned follow-up after 5 days, the patient was readmitted because of clinical worsening and an elevated CRP of 100 mg/L. The antibiotic treatment was changed to meropenem. More results from the BALF showed a positive PCR for SARS-CoV-2 with a Ct value of 32. After 3 days of meropenem, no improvement was seen and the CRP value continued to increase (126 mg/L), as did the liver enzymes (ALP 17 u/L [normal range 0.60–1.8 u/L], ALT 3.8 u/L [normal range 0.15–0.75 u/L]). With the medical history of the first patient (Case 1) in mind, a persistent SARS-CoV-2 infection was now suspected. The patient was started on nirmatrelvir/ritonavir for 5 days, after which fever, CRP, and liver enzymes rapidly decreased. After 5 days, CRP was almost normal (Figure 2) and the patient was discharged. Liver function tests were normalized after about 2 weeks. At the most recent follow-up (i.e. 6 months after discharge), the patient was still feeling well and reported resolution of all symptoms (Figure 2).

## Case 3

Case 3 was a 76-year-old male with a history of follicular lymphoma grade II, diagnosed in June 2021. Since November 2021, the patient had been treated with rituximab once a month, lenalidomide daily for 3 weeks with a 1-week pause, and epcoritamab once a month. He had received four doses of the SARS-CoV-2 vaccine, the last dose in March 2022, but had not responded serologically. Serum immunoglobulins had been tested before and were low with IgG at 3.2 g/L (normal range 6.7–14.5 g/L), IgA at 0.38 g/L (normal range 0.88–4.5 g/L), and IgM at 0.08 g/L (normal range 0.27–2.1). As he did not have any problems with infections, he was not on any immunoglobulin treatment. In April 2022, he developed a fever and respiratory symptoms and tested positive for SARS-CoV-2 by PCR in the nasopharynx but negative in blood. The strain was typed to BA.2. He was admitted to the hospital after 8 days and was treated with one dose of 300 mg tixagevimab-cilgavimab and remdesivir for 5 days. Because of a high CRP level (highest 133 mg/L), he received piperacillin-tazobactam followed by meropenem. He also received dexamethasone 6 mg for 5 days because of the elevated CRP and disease duration of >7 days. He did not require oxygen therapy. After a few days, he improved and was discharged after 9 days. He restarted the lymphoma treatment after 2 weeks with lenalidomide and epcoritamab as planned every month but rituximab was discontinued. During spring, summer, and autumn, he presented with a fever and respiratory symptoms about 1 week after every monthly epcoritamab treatment. Each time, he was prescribed different oral antibiotics (e.g. amoxicillin/clavulanic acid, doxycycline, or clindamycin). CRP was around 60–100 mg/L on all these occasions. Cultures from the nasopharynx and sinus were performed but with no positive findings. No viral PCR was taken during that time. In September, the Infectious Disease Clinic was consulted regarding the recurring infections and his low immunoglobulin levels (IgG 2.7 g/L, normal range 6.7–14.5 g/L). The next time (October 2022) the patient contracted a fever and respiratory symptoms, a PCR for respiratory viruses was taken, which was positive for SARS-CoV-2. At that time, nirmatrelvir/ritonavir was available in Sweden, and he was treated with that medication for 5 days. A SARS-CoV-2 serology test showed remaining anti-spike antibodies at 476 BAU/mL. The strain was typed to BA.2, which at that time in Sweden was an almost non-existent variant that strongly suggested prolonged SARS-CoV-2 infection with BA.2 rather than reinfection. A new SARS-CoV-2 PCR was taken after 3 weeks to determine whether the infection was cleared. It showed a Ct value of 21, and because the patient experienced clinical worsening with more cough, he was treated with another course of nirmatrelvir/ritonavir for 5 days and tixagevimab-cilgavimab 600 mg. The Ct values taken 1 and 2 weeks after treatment were 27 and 25, respectively. The patient initially experienced clinical improvement, but after 1 week, the symptoms returned, and he was reassessed at the Infectious Disease Clinic. The CRP was now normal (2 mg/L). After discussing with several Infectious Disease specialists and reviewing the scientific literature, the patient was treated with a combination of nirmatrelvir/ritonavir and remdesivir for 10 days. After that, the symptoms resolved, and during follow-up (the latest in May 2023), he continued to have no fever or respiratory symptoms and is considered cured of his persistent infection.

## Case 4

Case 4 was a 52-year-old female with a history of kidney transplantation (1992 and 2019) because of granulomatosis with polyangiitis (GPA). She was treated with rituximab every 6th month, 5 mg prednisolone, and 100 mg cyclosporine daily. She had received five doses of the SARS-CoV-2 mRNA vaccine but had not responded serologically. Serum immunoglobulins had been tested before and IgG was known to be decreased at 3.2 (normal range 6.7–14.5) but IgA and IgM were normal. The patient was not on any immunoglobulin treatment as she had not had any problems with infections during the last years. In November 2022, she developed a fever and respiratory symptoms. She was assessed at the Infectious Disease Clinic and tested positive for SARS-CoV-2 by PCR. The Ct value was 27 and CRP was 31 mg/L. PCR in blood was negative. Nirmatrelvir/ritonavir was discouraged as a therapy due to its interaction with cyclosporine. Thus, due to limited clinical symptoms, the patient was only treated with tixagevimab-cilgavimab 600 mg. The SARS-CoV-2 strain was later typed to BA.5. A few days later, she was reassessed at the Infectious Disease Clinic because of clinical deterioration with fever and worsening cough. CRP was now 47 mg/L, and with regard to her immunosuppressive therapy, she was prescribed amoxicillin orally. Two days later, she was admitted to the hospital because of a high fever, an elevated respiratory rate and CRP 67 mg/L but no supplemental oxygen requirement. The patient was started on piperacillin-tazobactam. A chest CT scan on day 18 after disease onset showed opacities consistent with SARS-CoV-2 infection. After admission, CRP and the fever initially decreased. An increase in liver enzymes was seen, which was determined to be caused by the piperacillin-tazobactam treatment, as the patient had a similar reaction when treated earlier with that antibiotic. After 9 days at the hospital, she was discharged. However, at the 1-week follow-up after discharge, she still had a fever and cough. A new SARS-CoV-2 PCR showed a Ct value of 28 and CRP had increased to 103 mg/L. The fever continued fluctuating, and the patient was finally treated with remdesivir for 5 days. The SARS-CoV-2 PCR in blood was negative. After the remdesivir treatment, the fever and respiratory symptoms resolved and CRP decreased to 50 mg/L (Figure 4). At the most recent follow-up in April 2023, she still had sustained resolution of symptoms.

## Discussion

Persistent SARS-CoV-2 infection in immunocompromised patients may be difficult to recognize and treat for the practicing clinician. We have presented four cases of patients with B-cell–depleting therapies who experienced long symptom durations (45–242 days) before the condition was recognized and treated. Fortunately, all patients finally responded to antiviral treatment and cleared the infection. However, one of the patients responded poorly to the initial antiviral treatment but eventually cleared the infection after experimental treatment with nirmatrelvir/ritonavir in combination with remdesivir.

Patients treated with rituximab have an impaired B-cell function that may lead to reduced vaccine response and increased susceptibility to infections (4). For SARS-CoV-2, the vaccine response rate after two doses is only about 30–40% (5, 6). All four cases had been vaccinated with several mRNA vaccines against SARS-CoV-2, but none responded serologically. Cases 1 and 3 had the strain BA.2 of the omicron variant, and both received the monoclonal antibody treatment tixagevimab-cilgavimab. Case 1 received this treatment late in the disease course together with nirmatrelvir/ritonavir and cleared the infection afterward. Case 3 received it early in the disease course (day 8) together with remdesivir but did not clear the infection. This patient was on a more potent immunocompromising treatment (rituximab plus epcoritamab), affecting both B- and T-cells. One can speculate that this therapy influenced the difficulty of clearing the infection. Cases 2 and 4 received tixagevimab-cilgavimab early in the disease course, but both had the strain BA.5 known to be much less sensitive to this monoclonal antibody treatment (11, 12). However, these patients received the antibody treatment before the actual strain was known.

The impact of time since the last rituximab treatment on the chances of clearing the SARS-CoV-2 infection has been discussed in the literature. One case report described a patient previously treated with rituximab who developed a persistent SARS-CoV-2 infection (3). In that report, the patient’s full recovery was not achieved until 7 months after the last rituximab dose when the autologous B-cell function had improved (3). This observation is consistent with our case series. Cases 1 and 3 received rituximab just days before disease onset and did not clear the infection until several months after inception. For cases 2 and 4, the rituximab treatments were given several months before the disease started, and they both cleared the infection more rapidly. However, for cases 1 and 3, the time from onset of disease to diagnosis was much longer than in cases 2 and 4, which certainly prolonged the disease duration. It is therefore difficult to determine how much the timing of rituximab treatment to disease onset has affected the disease duration of our patients. Still, it is an interesting suggestion worth further investigation.

It is essential to consider SARS-CoV-2 sampling from different locations in this patient group to increase diagnostic accuracy. Conventional sampling methods (nasopharynx PCR) may have reduced sensitivity because of lower viral load in the upper airways (3). Hence, sampling from lower airways and serum can be considered when the diagnosis is difficult. In cases 1 and 2, the SARS-CoV-2 infection would never have been confirmed without PCR from BALF, which was positive in contrast to repeated negative PCR samples from the nasopharynx. Ertesvåg et al. (3) described a similar case in which the patient was negative in the nasopharynx but positive in BALF.

Only a few reports have described disease courses and treatment strategies for B-cell–depleted patients with persistent SARS-CoV-2 infection, and even fewer in which a combination of antiviral treatments have been used. The first combination treatment was reported by Trottier et al. (10), where a B-cell–depleted patient was treated with remdesivir combined with nirmatrelvir/ritonavir for 20 days as neither remdesivir nor nirmatrelvir/ritonavir alone had cleared the infection. That patient was treated with the anti-CD20 monoclonal antibody obinutuzumab and cleared the infection after experimental dual antiviral therapy. This report led us to treat patient 3, who did not respond to single antiviral treatment. Later, three case reports (13–15), one case series (16), and a smaller study (17) were published in which combinations of antiviral drugs against SARS-CoV-2 were used to treat persistent infection in B-cell–depleted patients. In many cases, a single treatment was initially used but changed to combination treatment when the patient did not respond. Various combinations of remdesivir, nirmatrelvir/ritonavir, and molnupiravir have been used, with remdesivir plus nirmatrelvir/ritonavir being the most common combination. Different treatment durations have also been used for various drugs. One patient in the other case series was also treated with epcoritamab (16). In most of these cases, patients have also received monoclonal antibody treatment against SARS-CoV-2 and antiviral treatment. These publications and case 3 in our series suggest that dual antiviral treatment may be considered in patients with persistent SARS-CoV-2.

All patients in our case series received several courses of antibiotics, in one patient possibly resulting in elevated liver enzymes. Broad-spectrum antibiotics are often used when immunocompromised patients are suspected of infection. However, these treatments may also have side effects, including the risk of antibiotic resistance, disturbed host microbiota, and *Clostridioides difficile* infection. Early recognition of persistent SARS-CoV-2 infection is challenging but is of great importance for the patients because treatment and viral clearance might significantly improve quality of life and reduce unnecessary antibiotic treatments. The patients described in this case series had up to 230 days of viral shedding until the condition was finally recognized. To our knowledge, this is the longest viral shedding reported. Attempts have been made to better define the condition with persistent SARS-CoV-2 infection in immunocompromised patients. Dioverti et al. (2) proposed criteria for protracted COVID-19, while Belkin et al. (1) suggested similar criteria for the persistent inflammatory seronegative COVID syndrome. Both suggestions have the virologic criterium persistent or intermittent positive SARS-CoV-2 PCR for over 21 days in immunocompromised patients, where Belkin et al. focused mainly on B-cell deficiency and Dioverti et al. included B-cell deficiency and other immunodeficiencies. We agree with these authors that the condition needs to be better defined to achieve a scientific basis for recognition and future treatment guidelines.

To summarize, patients with B-cell deficiencies are at risk of prolonged SARS-CoV-2 infection, where short treatment courses with remdesivir or nirmatrelvir/ritonavir may not be sufficient as a treatment option. Persistent SARS-CoV-2 is a condition that can be challenging to diagnose and treat, and the awareness of this condition needs to be increased among clinicians treating immunocompromised patients. Moreover, proper diagnostic criteria for this condition must be established to promote better research. Once diagnosed with this condition, it appears to be treatable with antivirals even late in the disease course. However, which antivirals are best suitable for this treatment is not yet determined. Some immunocompromised patients do not respond to a single antiviral treatment against SARS-CoV-2. In these patients, a combination of drugs can be tested. However, which combinations and treatment durations are the most effective remains to be determined.
